# The Fate of Emotional Memories Over a Week: Does Sleep Play Any Role?

**DOI:** 10.3389/fpsyg.2019.00481

**Published:** 2019-03-05

**Authors:** Nicola Cellini, Marco Mercurio, Michela Sarlo

**Affiliations:** ^1^Department of General Psychology, University of Padua, Padua, Italy; ^2^Neuroscience Center, University of Padua, Padua, Italy

**Keywords:** actigraphy, arousal, emotional memory, sleep, valence, time

## Abstract

Although there is a wide consensus on how sleep processes declarative memories, how sleep affects emotional memories remains elusive. Moreover, studies assessing the long-term effect of sleep on emotional memory consolidation are scarce. Studies testing subclinical populations characterized by REM abnormalities are also lacking. Here we aimed to (i) investigate the fate of emotional memories and the potential unbinding (or preservation) between content and affective tone over time (i.e., 1 week), (ii) explore the role of seven nights of sleep (recorded via actigraphy) in emotional memory consolidation, and (iii) assess whether participants with self-reported mild-moderate depressive symptoms forget less emotional information compared to participants with low depression symptoms. We found that, although at the immediate recognition session emotional information was forgotten more than neutral information, a week later it was forgotten less than neutral information. This effect was observed both in participants with low and mild-moderate depressive symptoms. We also observed an increase in valence rating over time for negative pictures, whereas perceived arousal diminished a week later for both types of stimuli (unpleasant and neutral); an initial decrease was already observable at the immediate recognition session. Interestingly, we observed a negative association between sleep efficiency across the week and change in memory discrimination for unpleasant pictures over time, i.e., participants who slept worse were the ones who forgot less emotional information. Our results suggest that emotional memories are resistant to forgetting, particularly when sleep is disrupted, and they are not affected by non-clinical depression symptomatology.

## Introduction

In the last two decades, several studies have shown that sleep plays an important role in memory processing (Rasch and Born, 2013), including emotional memory, which can be defined as the memory of an event or experience that evokes an emotional response (Kensinger, 2009). According to the active system consolidation model (Diekelmann and Born, 2010), information initially encoded during wakefulness is repeatedly reactivated and reorganized during the subsequent sleep period in order to transform the labile memory trace into long-lasting memories. A key role in this process seems to be played by non-rapid eye movement (NREM, composed by N1, N2, and N3 stages) sleep, whose physiology seems to promote the reactivation of specific information at the hippocampal level and its reorganization and consolidation at cortical level (Staresina et al., 2015; Klinzing et al., 2016). However, while there is a wide consensus on how sleep, especially NREM sleep, processes declarative memory and, to a certain degree, procedural memories, how sleep affects emotional memories remains elusive (Ackermann and Rasch, 2014; Cellini and Capuozzo, 2018).

Although some recent studies have suggested a key role of NREM sleep in emotional memory processing (Hauner et al., 2013; Cellini and Parma, 2015; Diekelmann and Born, 2015; Cellini et al., 2016), most of the studies have focused on the role of REM sleep (Tempesta et al., 2017) due its unique state characterized by the suppression of adrenergic activity coupled with a large activation in the amygdala-hippocampal networks (Genzel et al., 2015; Pace-Schott et al., 2015). However, as recently highlighted by Bolinger et al. (2018), two contrasting hypotheses have been put forward about the role of REM sleep in emotional processing.

The first hypothesis, which is based on the “Sleep to Forget and Sleep to Remember” model (SFSR; Walker and van Der Helm, 2009), proposes that sleep depotentiates the emotional tone of experience while preserving the content of that event. Therefore, this model suggests that over several REM cycles the emotional tone (e.g., indexed by neural, peripheral, and subjective arousal) should decrease. The second hypothesis (“emotional salience view”) stems from studies showing a conservation of the emotional content after a sleeping period (Pace-Schott et al., 2011; Baran et al., 2012; Werner et al., 2015; Cellini et al., 2017) suggesting that REM sleep may instead preserve the emotional component of an experience during the consolidation of its content. In a recent review of the literature, Genzel et al. (2015) tried to merge these two hypotheses suggesting that REM sleep may promote the optimal process (preservation or reduction) depending on the nature of the information to be processed, in order to promote the most adaptive behavioral response during the next wakefulness.

The main issue about these models is that the majority of studies trying to test them have used a single-night (or nap) designs that could be insufficient to entirely capture the critical modulating role of sleep in emotional processing over time. Indeed, it seems plausible that several sleeping periods are needed to unbind the content and the emotional experience of an event. Moreover, given the focus of these models on the REM sleep, it is surprising that studies with clinical and subclinical populations characterized by abnormal REM sleep, such as narcolepsy (Cellini, 2017) or depression (Pillai et al., 2011; Palagini et al., 2013) are lacking. To our knowledge, there are only two studies, from the same group, investigating the relationship between sleep and emotional memory in a subclinical population with depressive symptoms (Harrington et al., 2018a,b). In one study, the authors tested two groups of participants with either minimal or mild-to-moderate depressive symptoms [based on the Beck Depression Inventory (BDI-II; Beck et al., 1996) scores] on a memory recognition task with emotional pictures and a within-subjects split-night design (Harrington et al., 2018a). In this paradigm, memory testing occurred after an N3-rich sleep in the first half of the night or a REM-rich sleep in the second half of the night, allowing them to differentiate (although with some limitations due to circadian and fatigue confounds) the role of N3 and REM sleep in emotional memory processing. They found a benefit for N3 sleep on neutral stimuli, and a marginal benefit for REM sleep on unpleasant pictures in the participants with mild-to-moderate depressive symptoms compared to the minimal depressive symptoms group. Also, they showed that participants with mild-to-moderate depressive symptoms had a greater REM density (number of rapid-eye movements/REM sleep duration), and a marginally greater number of rapid-eye movements in the second half of the night compared to the minimal depression group. In addition, the number of rapid-eye movements and REM density were positively associated with the discrimination of unpleasant pictures at the delayed recognition session. In the other study (Harrington et al., 2018b), the authors used the same groups and the same task but with a different protocol: participants underwent either a 12-h sleep or 12-h sleep deprivation between the encoding and the delayed recognition sessions. This time they found no differences in emotional memory consolidation between the two groups and no association between REM sleep and the consolidation of memories of unpleasant pictures. However, participants with mild-to-moderate depressive symptoms showed a lower memory retention in the sleep deprivation condition compared to the normal sleep condition for negative and neutral images (but not positive), whereas the minimal depressive symptom group showed no differences in the consolidation of negative and neutral images across conditions (although sleep deprivation impaired their ability to consolidate positive images). Whilst these findings suggest that emotional memory processing during sleep is affected by depressive symptomatology, further research is needed on this topic.

Based on this literature, here we aimed to (i) investigate the fate of emotional memories and the potential unbinding (or preservation) between emotional content and affective tone over time (i.e., 1 week); (ii) explore the impact of seven nights of sleep (recorded via actigraphy) on emotional memory consolidation; and (iii) assess whether participants with self-reported mild-moderate depressive symptoms forget less emotional memory information compared to participants with low depressive symptoms.

## Materials and Methods

### Participants

Forty-eight university students (27 F, Mean age ± Standard Deviation = 23.10 ± 2.53 years) participated in the study. All participants were enrolled through advertisements posted at the University of Padua and they underwent an online screening to ensure they met the eligibility criteria for the study. Exclusion criteria for all participants were the presence of psychiatric (e.g., clinical depression, anxiety disorders) or somatic diseases as evaluated by the screening questionnaires.

Based on their BDI-II scores, they were divided into lower depressive symptoms (LDS; *N* = 30) and higher depressive symptoms (HDS; *N* = 18) groups. The LDS group had BDI-II scores lower than 13 (i.e., the Italian BDI-II cut-off for minimal depression; Ghisi et al., 2006), whereas the HDS group had BDI-II scores between 13 and 28 (i.e., mild-moderate depression).

The study protocol was approved by the Ethics Committee of the Department of Psychology, University of Padua, and all the participants signed a written informed consent before participating in the study.

### Self-Reported Questionnaires

#### Pittsburg Sleep Quality Index (PSQI)

The level of self-reported sleep disturbances was assessed using the Pittsburg Sleep Quality Index (PSQI), a widely used questionnaire composed of 19 items (Buysse et al., 1989; Mollayeva et al., 2016). The scores range from 0 to 21, with 0 indicating no difficulties and 21 severe sleep difficulties. The commonly used cut-off to differentiate good from bad sleepers is >5 (Buysse et al., 1989; Curcio et al., 2013; Mollayeva et al., 2016).

#### Beck Depression Inventory-II (BDI-II)

The Beck Depression Inventory-II (BDI-II) is a 21-item questionnaire to assess the severity of depressive symptomatology (Beck et al., 1996). The total score ranges from 0 to 63, with the higher scores indicating more severe depressive symptoms. For the Italian version of the BDI-II, a score of 13 is considered as the optimal cut-off to discriminate individuals with and without depressive symptoms (Ghisi et al., 2006).

#### State-Trait Anxiety Inventory Y2 (STAI-Y2)

The trait anxiety level was assessed using the State-Trait Anxiety Inventory version Y2 (STAI-Y2) (Spielberger, 2010). This self-report questionnaire is composed of 20 items, with a total score ranging from 20 to 100. Higher scores indicate greater anxiety levels.

#### Circadian Preferences

Circadian preferences were assessed using the reduced version of morningness–eveningness questionnaire (MEQr, Adan and Almirall, 1991), translated into Italian (Natale, 1999; Natale et al., 2006a,b). This questionnaire assesses self-reported chronotype using 5 items, with a total score ranging from 4 to 35, which categorize participants into evening (scores <11), intermediate (scores between 11 and 18), and morning types (scores >18).

### Emotional Memory Task

All the participants performed an emotional memory task divided into encoding and two recognition sessions (immediate and delayed recognition sessions). One-hundred and sixty digitized pictures were selected from the International Affective Picture System (IAPS) (Lang et al., 2008) based on their normative arousal and valence ratings and were organized in four sets of 40 pictures, each composed of 20 unpleasant stimuli (attacking humans and animals, injuries, and mutilations; mean normative ratings: arousal 5.75, valence 2.71) and 20 neutral stimuli (household objects, neutral faces and urban landscapes; mean normative ratings: arousal 3.11, valence 4.96). The sets were balanced in terms of normative valence and arousal ratings (see Supplementary Material). The presentation of the sets and the sequence of the blocks within each set were counterbalanced across participants using a Latin square design.

**FIGURE 1:**
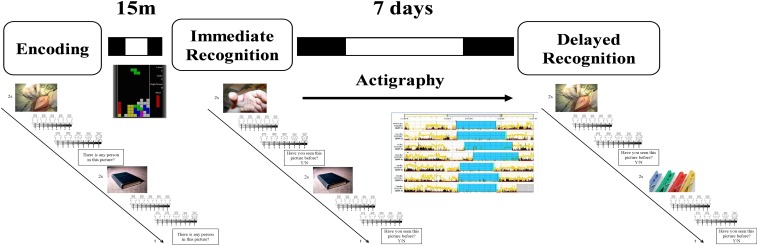
Schematic representation of the experimental procedure. During the encoding session, participants were exposed to 40 neutral (objects, neutral faces) and 40 negative (injuries, attacking humans, and animals) pictures. They were asked whether a person was present in the picture or not. After each picture they rated how they felt in term of valence and arousal. After the task they played Tetris for 15 min before performing an immediate recognition session. During the immediate (15 min later) and the delayed (7 days later) recognition task, half of the pictures previously presented were randomly rearranged and intermixed with 40 new pictures similar in term of content, and normative valence and arousal ratings. Participants had to decide whether they had seen or not the picture in the previous session. Between the immediate and the delayed recognition sessions participants wore an actigraph and complete a daily sleep log.

During the encoding task (Figure 1), participants were exposed to a set of 40 neutrals and 40 unpleasant pictures. At the beginning of each trial, a gray cross (+) appeared at the center of a black screen (duration: 1 s) followed by the presentation of one of the pictures for 2 s. After that, participants were asked to decide (no time limit) whether in the pictures there were one or more persons or not, by pressing the “A” or the “L” button, respectively, on a QWERTY keyboard. This request was aimed to force participants to attend to the features of the images, and also as a check to ensure that the participants remained focused during the task. Afterward, participants rated their subjective valence (i.e., state of pleasantness) and arousal (i.e., state of activation) evoked by picture viewing using two 9-point graphic scales (from 1 to 9) of the computerized version of the Self-Assessment Manikin (Lang et al., 2008). The 80 pictures were presented in a random order for all the participants.

In both the immediate and the delayed recognition tasks, participants were exposed again to 80 pictures, 40 already presented in the encoding task (20 neutral and 20 unpleasant) intermixed with 40 new pictures (20 neutral and 20 unpleasant). In this case, after picture presentation, participants had to decide whether they had already seen the pictures before or not, by pressing the “A” or the “K” button, respectively. The task was implemented and run with E-Prime 2.0 (Psychology Software Tools, Inc., Pittsburgh, PA, United States).

Performance in this task was computed using signal detection theory (Macmillan and Creelman, 2004). For each participant and for each recognition session, we computed the following variables separately for neutral and unpleasant stimuli: Hits (i.e., the number of old pictures correctly defined as seen), Correct Rejections (i.e., the number of novel pictures correctly defined as never seen), Misses (i.e., the number of old pictures mistakenly defined as never seen), and False Alarms (i.e., the number of novel pictures mistakenly defined as seen). From these variables we calculated the Hit Rate (HR), the False Alarm Rate (FAR), and the memory discrimination index (*d*’) as the difference between the z-transformed (normalized) probabilities of Hit Rate and False Alarm Rate [*d*’ = z(HR) – z(FAR)]. Since *d*’ cannot be calculated when HR = 1 or FAR = 0, we replaced HR values of 1 and FAR values of 0 with 1 – 1/(2N) and 1/2N, respectively, where N equals the number of targets (Macmillan and Creelman, 2004).

### Actigraphic Recording

Participants’ sleep patterns were assessed using the Actiwatch-64 (AW-64; Philips Respironics, Portland, OR, United States), a reliable actigraph to objectively measure sleep parameters based on the level of physical activity (Cellini et al., 2013). Actigraphic data were collected for 7-days in 1-min epochs. Participants were instructed to continuously wear the actigraph on the non-dominant wrist and to press the AW-64 marker button every time they switched off/on the light to sleep and to get up from the bed, or when they had to remove the AW-64 for any reason (e.g., coming in contact with water). Actigraphic data were analyzed using the Actiware 6.2 software (Phillips Respironics, Portland, OR, United States), using the Medium threshold setting (40 activity counts/epoch to define a wake state). All analyses were confined to the period between lights off and lights on, which was defined based on both the presence of marker placement and bedtimes and waketimes reported by the participants in their sleep diary. For each participant and for every night we calculated the total sleep time (TST, min), defined as the number of minutes scored as sleep between lights off and lights on; sleep onset latency (SOL, min), the number of minutes between lights out and the first epoch scored as sleep; wake after sleep onset (WASO, min), the number of minutes scored as WASO; and sleep efficiency (SE, %), the ratio between TST and total time spent in bed.

### Procedure

Participants completed online a battery of questionnaires including the BDI-II, the PSQI, the STAI-Y2, and the rMEQ. Then, participants were scheduled for the experimental session in the lab where they first performed the encoding session and, 15 min after the end of the encoding, they had the immediate recognition session (Figure 1). Between the encoding and the immediate recognition sessions, participants played with a freely available version of Tetris in order to avoid active rehearsal of the pictures. Before leaving the lab, participants received a sleep diary, a wrist actigraph, and the instruction to use these instruments during the following 7 days. A week later, they returned to the laboratory at the same time as the week before to give back the sleep diary and the actigraph, and to perform the delayed recognition session.

### Statistical Analysis

Demographics and sleep variables were compared between the two groups using independent *t*-tests and χ^2^ for continuous and categorical data, respectively.

Separate 2 × 2 × 2 ANOVAs with *Group* (LDS, HDS) as a between-subject factor, and *Category* (Unpleasant, Neutral) and *Session* (Immediate, Delayed) as within-subject factors were run to assess group differences in the memory parameters (i.e., Schematic representation, HR, FAR).

For the analysis of the self-rated arousal and valence, we calculated separately the mean valence and arousal ratings from the encoding session for images that subsequently appeared in the immediate and delayed recognition test (“old” images). Then we analyzed these data with separate 2 × 2 × 2 ANOVAs with *Group* (LDS, HDS) as a between-subjects factor and *Category* (Unpleasant, Neutral) and *Session* (Encoding, Immediate, or Delayed Recognition Test) as within-subject factors for the self-rated arousal and valence. This approach, focusing only on target images, allowed us to assess the presence of any habituation effect due to the mere re-exposure to similar images (immediate recognition test) or to slower emotional processing that may occur across the 7-day interval.

Fisher’s Least Significant Difference test was used for *post hoc* comparisons and partial eta squared ($\eta_{p}^{2}$) was reported as estimate of effect size.

Pearson’s correlations were run to explore potential associations between sleep parameters averaged across the week (see Table 1) and the change (computed as delayed minus immediate test score) in behavioral variables (d’, HR, FAR, arousal, and valence).

**Table 1:** Demographics, psychological measures, and sleep parameters of the sample.

	LDS		HDS				
	Mean	*SD*	Mean	*SD*	*t*_(45)_	*p*	Cohen’s *d*
**Demographics and baseline measures**							
Age	23.32	3.01	22.74	1.46	0.77	0.443	0.23
Gender (F/M)	19/11		8/10		1.63^∗^	0.20^∗^	
BDI-II	5.93	3.72	18.72	4.74	-10.39	<0.001	-3.10
STAI-Y2	38.47	8.38	56.56	10.65	-6.54	<0.001	-1.95
PSQI	5.67	2.06	7.82	2.63	-3.12	0.003	-0.95
MEQr	14.00	2.65	13.29	4.25	0.70	0.490	0.21
**Sleep parameters**							
Bed time (hh:mm)	1:02	00:59	1:16	1:15	-0.65	0.517	-0.20
Wake time (hh:mm)	8:43	00:59	8:41	00:44	0.12	0.906	0.04
Time in bed (min)	454.81	36.99	445.16	48.75	0.71	0.483	0.21
Sleep latency (min)	6.62	7.96	11.24	5.64	-2.32	0.025	-0.70
WASO (min)	49.82	23.53	43.20	26.02	0.87	0.391	0.26
Total sleep time (min)	398.37	48.22	390.72	47.34	0.53	0.600	0.16
Sleep efficiency (%)	87.55	6.10	87.69	5.71	-0.08	0.938	-0.02


*BDI-II, Beck Depression Inventory-II; STAY-Y2, State-Trait Anxiety Inventory Y2; PSQI, Pittsburg Sleep Quality Index; MEQr, reduced version of morningness–eveningness questionnaire; WASO, wake after sleep onset. ^∗^χ^2^ value.*

## Results

Descriptive statistics of the sample are presented in Table 1.

The HDS group showed a higher BDI-II score, reflecting the selection criteria, as well as higher STAI-Y2 and PSQI scores, which are usually positively associated with BDI-II scores (e.g., Demirci et al., 2015; Mollayeva et al., 2016; Çelik et al., 2018). At the objective sleep level, the only differences were observed for the sleep onset latency: on average, HDS took longer to fall asleep than LDS. No other significant difference was observed. Comparing objective (actigraphy) and subjective (sleep diary) sleep parameters, we observed longer reported time spent in bed, asleep, trying to fall asleep and higher sleep efficiency for the subjective measures (see Supplementary Material for the full statistics). This is likely due to the tendency of the participants to “round up” the reported time (e.g., instead of 7 h and 20 min spent in bed they tend to report 7 h and 30 min). Nevertheless, no differences between the two depressive symptom groups were observed for sleep diary parameters (all *p*’s > 0.29, Supplementary Table S2).

### Valence and Arousal Ratings

#### Immediate Recognition Test

**FIGURE 2:**
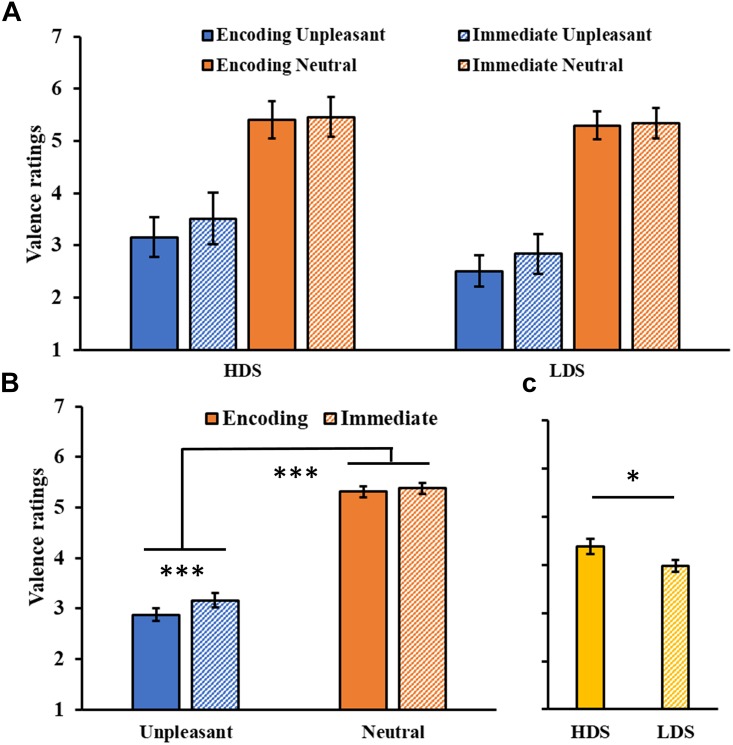
**(A)** Valence ratings as a function of the group and of the category of stimuli (unpleasant and neutral pictures) at the encoding and at the immediate recognition sessions. **(B)** Session (encoding and immediate recognition sessions) × Category (unpleasant and neutral pictures) interaction for the valence ratings. **(C)** Group (HDS and LDS) main effect for valence ratings. LDS: participants with lower depression symptoms; HDS: participants with higher depression symptoms. Error bars represent standard error of the mean. ^∗^*p* < 0.05; ^∗∗∗^*p* < 0.001.

The analysis on valence ratings (Figure 2A) showed a significant Session main effect (*F*_1,46_ = 19.23, *p* < 0.001, $\eta_{p}^{2}$ = 0.20), with a general increase in the valence of the images in the immediate test compared to the encoding session. A significant Session × Category interaction was found (*F*_1,46_ = 10.01, *p* = 0.003, $\eta_{p}^{2}$ = 0.18, Figure 2B), with a significant increase in valence for the unpleasant stimuli (i.e., they became less unpleasant) in the immediate test compared to the encoding session (*p* < 0.001). We also observed a significant Category main effect (*F*_1,46_ = 338.569, *p* < 0.001, $\eta_{p}^{2}$ = 0.88), with higher valence ratings for neutral than unpleasant pictures, and a Group main effect (*F*_1,46_ = 4.26, *p* = 0.045, $\eta_{p}^{2}$ = 0.08, Figure 2C), with a generally higher self-reported valence for the HDS group than the LDS group. This latter result seems to be driven specifically by the higher valence for unpleasant stimuli (i.e., less unpleasant) in the HDS compared to the LDS group (*p* = 0.005), even if the Group × Category interaction did not reach statistical significance (*F*_1,46_ = 3.41, *p* = 0.071, $\eta_{p}^{2}$ = 0.07).

**FIGURE 3:**
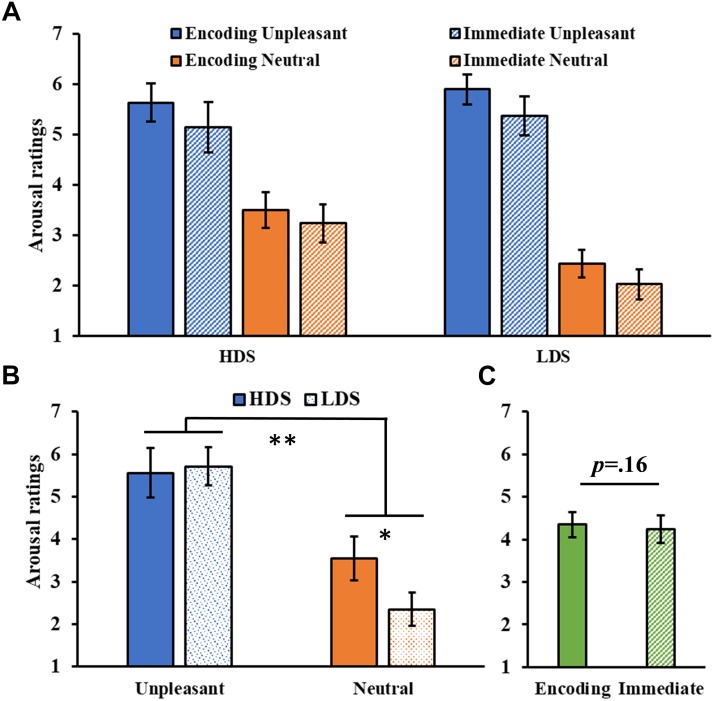
**(A)** Arousal ratings as a function of the group and of the category of stimuli (unpleasant and neutral pictures) at the encoding and at the immediate recognition sessions. **(B)** Group (HDS and LDS) × Category (unpleasant and neutral pictures) interaction for the arousal ratings. **(C)** Session (encoding and immediate recognition sessions) main effect for arousal ratings. LDS: participants with lower depression symptoms; HDS: participants with higher depression symptoms. Error bars represent standard error of the mean. ^∗^*p* < 0.05; ^∗∗^*p* < 0.01.

The analysis of the arousal ratings (Figure 3A) showed a significant Category main effect (*F*_1,46_ = 142.43, *p* < 0.001, $\eta_{p}^{2}$ = 0.76), with higher arousal ratings for unpleasant than neutral pictures, and a significant Group × Category interaction (*F*_1,46_ = 9.02, *p* = 0.004, $\eta_{p}^{2}$ = 0.16, Figure 3B), with the HDS group showing higher arousal scores for neutral pictures compared to the LDS group (*p* = 0.017). No main effect of Session was observed (*F*_1,46_ = 2.06, *p* = 0.16, $\eta_{p}^{2}$ = 0.04, Figure 3C).

#### Delayed Recognition Test

**FIGURE 4:**
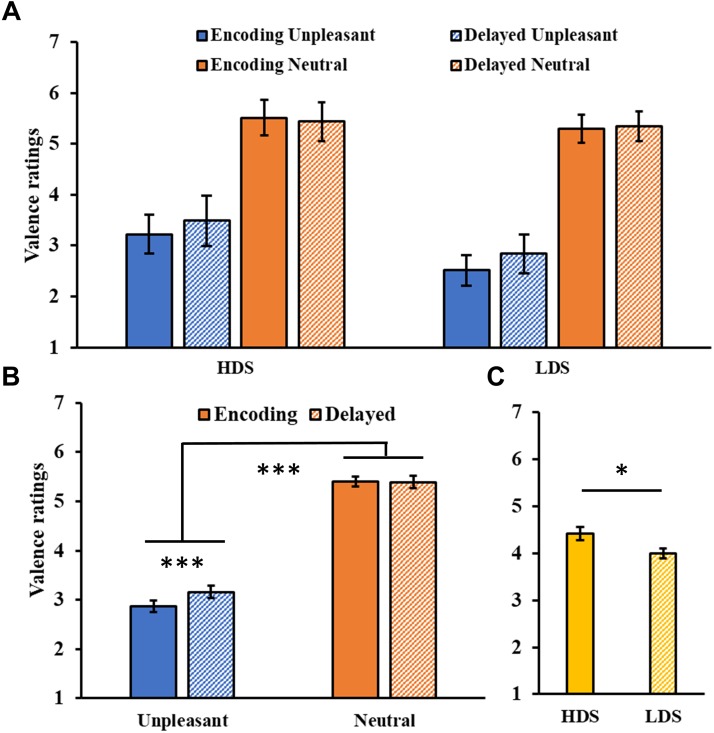
**(A)** Valence ratings as a function of the group and of the category of stimuli (unpleasant and neutral pictures) at the encoding and at the delayed recognition sessions. **(B)** Session (encoding and delayed recognition sessions) × Category (unpleasant and neutral pictures) interaction for the valence ratings. **(C)** Group (HDS and LDS) main effect for valence ratings. LDS: participants with lower depression symptoms; HDS: participants with higher depression symptoms. Error bars represent standard error of the mean. ^∗^*p* < 0.05; ^∗∗∗^*p* < 0.001.

The analysis of valence ratings (Figure 4A) showed a significant Session main effect (*F*_1,46_ = 6.21, *p* = 0.016, $\eta_{p}^{2}$ = 0.12), and a significant Session × Category interaction (*F*_1,46_ = 15.00, *p* < 0.001, $\eta_{p}^{2}$ = 0.25, Figure 4B), with a significant increase in valence for the unpleasant stimuli (i.e., they became less unpleasant) in the delayed recognition test compared to the encoding session (*p* < 0.001). The analysis also showed a significant Category main effect (*F*_1,46_ = 285.31, *p* < 0.001, $\eta_{p}^{2}$ = 0.86), with higher valence ratings for neutral than unpleasant pictures, and a Group main effect (*F*_1,46_ = 5.46, *p* = 0.024, $\eta_{p}^{2}$ = 0.11, Figure 4C), with a generally higher self-reported valence for the HDS group. Again, this latter result seems to be driven specifically by the higher valence for unpleasant stimuli (i.e., less unpleasant) in the HDS compared to the LDS group (*p* = 0.004), even if the Group × Category interaction did not reach statistical significance (*F*_1,46_ = 3.48, *p* = 0.068, $\eta_{p}^{2}$ = 0.07). No other significant differences were observed.

**FIGURE 5:**
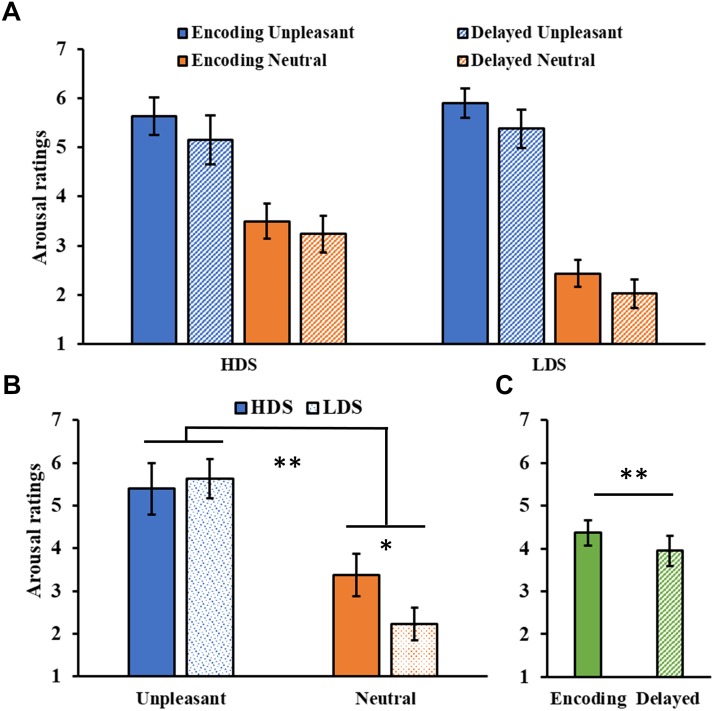
**(A)** Arousal ratings as a function of the group and of the category of stimuli (unpleasant and neutral pictures) at the encoding and at the delayed recognition sessions. **(B)** Group (HDS and LDS) × Category (unpleasant and neutral pictures) interaction for the arousal ratings. **(C)** Session (encoding and delayed recognition sessions) main effect for arousal ratings. LDS: participants with lower depression symptoms; HDS: participants with higher depression symptoms. Error bars represent standard error of the mean. ^∗^*p* < 0.05; ^∗∗^*p* < 0.01.

The analysis of the arousal ratings (Figure 5A) showed a significant Category main effect (*F*_1,46_ = 155.09, *p* < 0.001, $\eta_{p}^{2}$ = 0.77), with higher arousal ratings for unpleasant than neutral pictures. A significant Group × Category interaction (*F*_1,46_ = 10.01, *p* = 0.003, $\eta_{p}^{2}$ = 0.18, Figure 5B), revealed that the HDS group showed higher arousal scores for neutral pictures compared to the LDS group (*p* = 0.023). Unlike the immediate test results, this time we observed a main effect of Session (*F*_1,46_ = 9.67, *p* = 0.003, $\eta_{p}^{2}$ = 0.17, Figure 5C), with a general decrease in arousal for both category of stimuli in the delayed recognition test compared to the encoding session. No other significant differences were observed.

### Memory Performance

#### Encoding

During the encoding phase, participants detected the presence of one/more person with an accuracy of (Mean ± SD) 94.25 ± 4.96%, indicating a high level of attentional focus on the pictures presented on the screen.

#### Memory Discrimination (*d*’)

**FIGURE 6:**
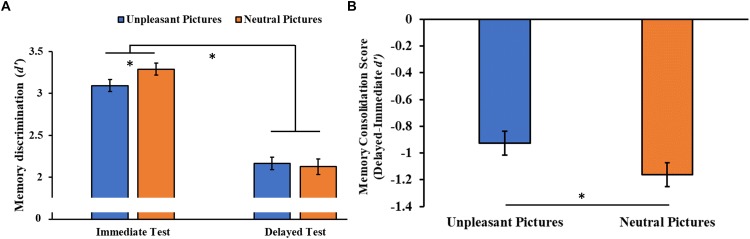
**(A)** Memory discrimination (*d*’) as a function of the testing session (immediate and delayed recognition sessions) and of the type of stimuli (unpleasant and neutral pictures). **(B)** Memory consolidation score (delayed minus immediate test *d*’ score) as a function of the type of stimuli. Error bars represent standard error of the mean. ^∗^*p* < 0.05.

The analysis of the consolidation of the stimuli over time (i.e., the discriminability of the encoding pictures in the immediate and delayed recognition sessions) showed a significant Session main effect (*F*_1,46_ = 246.85, *p* < 0.001, $\eta_{p}^{2}$ = 0.84), with *post hoc* tests indicating a lower memory discrimination index (*d*’) for the stimuli at the delayed compared to the immediate recognition session (*p* < 0.001). We also observed a significant Session × Category interaction (*F*_1,46_ = 4.08, *p* = 0.049, $\eta_{p}^{2}$ = 0.08, Figure 6A), with a higher memory discrimination index for neutral stimuli relative to unpleasant pictures at the immediate (*p* = 0.019), but not the delayed recognition session (*p* = 0.450), indicating less forgetting of the unpleasant stimuli. To confirm this result, we compared the memory consolidation score (i.e., delayed minus immediate *d*’ score) of the two stimuli category, showing the same result (*t*_96_ = 2.13, *p* = 0.036, Figure 6B).

#### Hit Rate and False Alarm Rate

**FIGURE 7:**
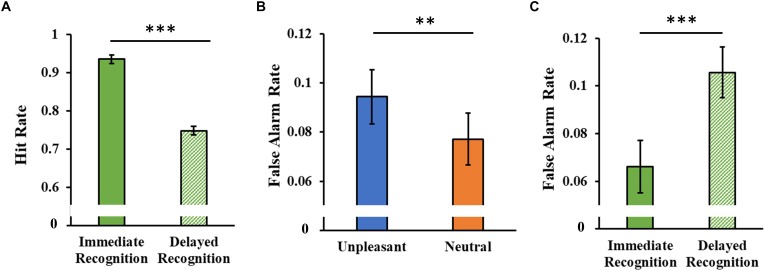
**(A)** Hit rate as a function of the experimental session (immediate and delayed recognition sessions). **(B)** False alarm rate as a function of the type of stimuli (unpleasant and neutral pictures). **(C)** False alarm rate as a function of the experimental session (immediate and delayed recognition sessions). Error bars represent standard error of the mean. ^∗∗^*p* < 0.01; ^∗∗∗^*p* < 0.001.

The analysis of the hit rate showed only a significant main Session effect (*F*_1,46_ = 115.97, *p* < 0.001, $\eta_{p}^{2}$ = 0.71), with a decrease in hit rate in the delayed session compared to the immediate one (Figure 7A).

The analysis of the false alarm rate showed a significant main Category effect (*F*_1,46_ = 8.08, *p* = 0.007, $\eta_{p}^{2}$ = 0.15, Figure 7B), with a higher false alarm rate for the unpleasant stimuli. A significant main Session effect (*F*_1,46_ = 12.60, *p* < 0.001, $\eta_{p}^{2}$ = 0.22) revealed an increased false alarm rate in the delayed session compared to the immediate one (Figure 7C).

### Correlational Analyses on Performance and Sleep Parameters

**FIGURE 8:**
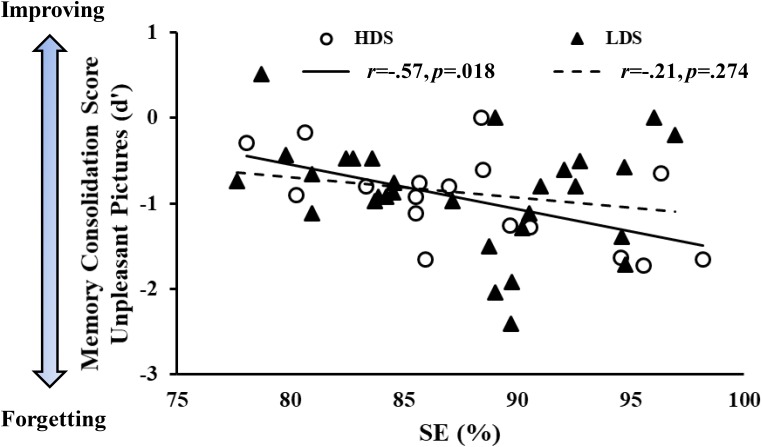
Memory consolidation score (*d*’ of the delayed minus *d*’ of the immediate recognition session) of the unpleasant pictures as a function of sleep efficiency (SE) in the two groups.

We observed a negative association between sleep efficiency (SE, %) and memory consolidation score of unpleasant stimuli over time in the HDS group (*r* = -0.57, *p* = 0.018), but not in the LDS group, which showed a similar pattern without reaching a statistical significance (*r* = -0.21, *p* = 0.274, Figure 8). This association was also present when the two groups were merged (*r* = -0.32, *p* = 0.026, Supplementary Figure S1), and by correcting the correlation of the whole sample for the BDI-II scores (partial correlation with BDI-II as covariate, *r* = -0.33, *p* = 0.026). These results indicate that in the participants with higher symptoms of depression, lower sleep quality was associated with less forgetting of emotional stimuli. Comparing the two correlation slopes with the Fisher’s r-to-z transformation (Cohen et al., 2003), we did not observe a significant difference between the groups (*Z* = -1.35; *p* = 0.18, two tails).

## Discussion

In the current study, we aimed to investigate the potential role of sleep in the consolidation of emotional memories over 7 days. Moreover, we wanted to assess emotional memory processing over time in a population at risk of REM abnormality, i.e., individuals with self-reported mild-moderate depressive symptoms.

We found that, although at the immediate recognition session emotional information was forgotten more than neutral information, a week later emotional information was forgotten less than neutral information. This effect was observed both in participants with low and mild-moderate depressive symptoms. We also observed an increase in valence ratings both at the immediate and at the delayed recognition session, suggesting a habituation effect to these stimuli. Perceived arousal decreased a week later (but not during the first re-exposure to the images, i.e., immediate session) for both types of stimuli (unpleasant and neutral), suggesting a potential role of sleep in the processing of all the stimuli. Interestingly, we observed a negative association between sleep efficiency across the week and change in memory discrimination for unpleasant pictures over time, i.e., participants who slept worse were the ones who forgot less (remembered more) emotional (unpleasant) information. This latter effect was significant for the participants with self-reported mild-moderate depressive symptoms, but the participants with low depressive symptoms showed the same trend.

Regarding memory performance, we observed, consistent with previous studies from our groups (Cellini et al., 2016; Mercurio et al., 2018) and others (Wagner et al., 2007; McKeon et al., 2012), that neutral stimuli are encoded better than the unpleasant ones. This result may seem unexpected given the wide literature about the memory advantage for emotional events (Kaplan et al., 2016). However, an explanation of this finding may be linked to the nature of the task and of the stimuli and the level of their arousal. We used a recognition task, which required encoding the details of the pictures presented (the IAPS pictures); these stimuli are known to reliably evoke an emotional response both at the subjective and physiological levels (Lang et al., 2008). Neutral pictures, due to their low arousal level, may be scanned profoundly, resulting in a better encoding of the picture details. Instead, highly arousing unpleasant pictures can only capture the attention of the participants on the general perspective of the picture (e.g., this is a black spider), leaving limited attentional resources to explore the details of the pictures (e.g., this spider has eight legs). When the participants are exposed to similar pictures (a black spider in a different position with four legs), the limited encoding of the details may increase the number of errors, i.e., the false alarm rate, as observed in the current and in previous studies (see Groch et al., 2014; Cellini et al., 2016). This effect may be similar, to some extent, to the emotional memory narrowing (Kensinger, 2009) and the weapon focus effect (Loftus et al., 1987), characterized by an attentional trade-off for central versus peripheral details of the emotional events (Kaplan et al., 2016).

Interestingly, over the week, the memory of the unpleasant stimuli decayed to a lesser degree, i.e., they were forgotten less. This result is intriguing since it suggests that details of emotional events may be harder than neutral to be encoded, but then they become resistant to forgetting. This result fits well with a study by Dolcos et al. (2005), which showed that memory for unpleasant pictures was greater than for neutral ones 1 year after encoding, and the memory benefit was driven by enhanced recollection (i.e., the process of retrieving contextual information about an event) rather than familiarity (i.e., the feeling of having experienced an event). Another study (Wagner et al., 2006) showed that the content of emotional memory (i.e., emotional texts) can be recognized up to 4 years after the encoding if the participants slept after the encoding. These results suggest that once the details of emotional events are correctly encoded, then this memory become more resistant to forgetting compared to neutral information.

One of the aims of the current study was to test the relationship between sleep and emotional memory in individuals with mild-moderate depressive symptoms. This idea stems from recent work by Harrington et al. (2018a,b), which was grounded in the observation that individuals with depression show marked changes in sleep pattern, for example, modification in the REM characteristics such as its latency, proportion, and density (Pillai et al., 2011; Palagini et al., 2013). Considering the theories postulating a key role of REM sleep in emotional memory processing (Walker and van Der Helm, 2009; Genzel et al., 2015; Tempesta et al., 2017), alterations in REM sleep should change the emotional processing, for example, by strengthening the salience of emotional information. However, similarly to the sleep condition in the second Harrington et al. (2018b), study here we did not observe any difference in emotional memory consolidation between the groups with different level of depressive symptomatology. Taken together, these results suggest that, at least in a non-clinical population, the level of depressive symptomatology may not affect emotional memory consolidation over time.

Nevertheless, we observed that participants with higher level of depressive symptomatology rated the unpleasant pictures as less unpleasant than the participants with lower BDI-II score, both at the immediate and at the delayed recognition session. Although this may seem the opposite of what one might expect to observe, it is in line with the *emotion context-insensitivity hypothesis* proposed by Rottenberg and Gotlib (2004) which suggests that individuals with depression tend to show less affective modulation (see Rottenberg et al., 2005). This seems to be related to dysfunctional emotional regulation, which makes the emotional response to stimuli more insensitive and less adequate to the context. Although Rottenberg et al. (2005) based their hypothesis on major depressive disorder, it is possible that participants with higher BDI-II scores may have experienced a similar insensitivity (at least to some extent) to the emotional pictures presented in the current study.

From a theoretical perspective, the current data can partially fit the different hypotheses concerning the role of sleep in emotional memory processing (Walker and van Der Helm, 2009; Pace-Schott et al., 2011; Baran et al., 2012; Werner et al., 2015). Emotional pictures were more likely to be retained than neutral pictures across the 7-day interval, which is in line with all the models suggesting a stronger long-term consolidation of emotional over neutral memories. Moreover, the valence ratings of the unpleasant pictures increased over time (i.e., they were perceived as less unpleasant). However, this effect was already present at the immediate recognition session, therefore questioning the role of sleep in reducing the unpleasantness of these memories. Also, while the SFSR hypothesis would predict an arousal decrement only for unpleasant pictures over a week (Walker and van Der Helm, 2009), and the emotional salience view would expect a preservation of the arousal ratings (Pace-Schott et al., 2011; Baran et al., 2012; Werner et al., 2015), here we observed a reduction of perceived arousal over a week for both types of stimuli. Moreover, whilst sleep was associated with memory forgetting, it did not play a critical role in modulating perceived arousal and valence ratings (as indicated by the lack of any correlation between sleep parameters and change in affective ratings). Overall, based on the results of the current study, whether sleep induced a modification in the emotional tone over a week (including several REM cycles, as proposed by the SFSR and the other theoretical models) remains unclear; the current results cannot clearly be interpreted within one of the current models regarding sleep and memory processing.

Nevertheless, our results demonstrate that sleep efficiency was linearly associated with memory forgetting. Although this result may seem counterintuitive, it should be interpreted in the context of a laboratory study. It is plausible that, over time, the brain would get rid of non-useful information (Davis and Zhong, 2017), such as the stimuli used in the current study, to make room for more salient information, in line with the synaptic homeostasis hypothesis (Tononi and Cirelli, 2014). However, as recently proposed by Feld and Born (2017), the sleeping brain sculpts memories by, on the one hand, actively deleting non-useful memory traces (such as neutral non-arousing stimuli) and, on the other hand, by protecting salient arousing information. However, it is plausible that the salience of a memory (useful or non-useful) may change across time, depending on how much individuals use that memory. Indeed, it has been shown that the protective effect of sleep seems to be a function of the time from the encoding to the retrieval, for example, it seems that there is a “protecting” effect of memories in the 24-h after the encoding, but then the sleep benefit begins to disappear (Schönauer et al., 2015; Abel et al., 2018), with forgetting following a log-linear curve over the next days (Murre and Dros, 2015). In the case of emotionally arousing stimuli, such as in the unpleasant pictures of the current study, we can speculate that sleep (at least nocturnal sleep, see Cunningham and Payne, 2017) may promote a stronger initial consolidation of these stimuli to the detriment of neutral ones. This will result in the normal decay for neutral information, which can reach a “plateau” level about 7 days after the encoding (Murre and Dros, 2015). Afterward, it seems that there is a second forgetting phase (Fisher and Radvansky, 2018). Instead, the forgetting curve of unpleasant stimuli may be delayed and it is plausible that a sleep-related forgetting mechanism may still be active about 7 days after the encoding. This delay may explain the positive relationship between sleep efficiency and forgetting observed only for unpleasant stimuli. Moreover, this delay may also be worsened in the HDS group due to potential impairment of neural plasticity, which has been reported in patients with major depressive disorder, and has been associated with maladaptive synaptic downregulation (Wolf et al., 2016). Nevertheless, it should be stressed that since we did not collect memory performance across the week (e.g., after 1–3–5 days after the encoding), this explanation should be considered as mere speculation.

The current results should be interpreted in the context of the study’s limitations. For example, we used actigraphy instead of polysomnography (PSG) to assess sleep pattern over a week. Although standard actigraphy is considered a reliable tool to objectively assess sleep, it also has some limits, such as low specificity (i.e., the ability to correctly detect wakefulness) and cannot differentiate across sleep stages, not allowing assessment of the potential role of REM sleep in emotional memory processing over a week. Therefore, the association between sleep efficiency over a week and memory retention should be considered with some caution. Further studies employing standard PSG, which allow not only the differentiation of sleep stages but also the analysis of micro-sleep architecture (e.g., sleep spindles, slow oscillation, theta activity), can eventually corroborate and extend the current findings. Also, in our sample the depressive symptoms were exclusively evaluated using a single questionnaire (the BDI-II) administered once, and the inclusion criteria for the minimal depression group were less severe, in contrast to the more comprehensive sample selection of Harrington and colleagues (Harrington et al., 2018a,b). Lastly, we cannot exclude that using a different paradigm (e.g., a recall task instead of a recognition task) and/or a different type of stimuli (e.g., stories, video clips) would result in different outcomes.

## Conclusion

In conclusion, we showed that while the self-reported valence and arousal changed as a function of the re-exposure to the emotional stimuli over time, emotional memories became resistant to forgetting, particularly when sleep was disrupted, and they were not affected by non-clinical levels of depression.

## Data Availability

The datasets generated for this study are available on request to the corresponding author.

## Author Contributions

NC and MS developed the study concept and contributed to the study design. NC and MM performed the testing, data collection, and data analysis. All authors interpreted the data, drafted the manuscript, provided critical revisions, and approved the final version of the manuscript for submission.

## Conflict of Interest Statement

The authors declare that the research was conducted in the absence of any commercial or financial relationships that could be construed as a potential conflict of interest.
