# Supplementary material for: The Fate of Emotional Memories Over a Week: Does Sleep Play Any Role?
Source: Front Psychol. 2019 Mar 5;10:481. doi: 10.3389/fpsyg.2019.00481 (PMC6411793; doi:10.3389/fpsyg.2019.00481)
Supplement: Supplementary file 1 [file Data_Sheet_1.PDF]

The fate of emotional memories over a week: does sleep play any role?

Nicola Cellini[1*], Marco Mercurio[1] & Michela Sarlo[1,2]

[1]Department of General Psychology, University of Padova, Padova, Italy

[2]Neuroscience Center, University of Padova, Padova, Italy

Supplemental Material

Normative arousal and valence rating of the four experimental sets

To test whether the four picture sets were balanced in term of normative valence and arousal, we ran two separate 2 (image category: unpleasant and neutral) x 4 (image sets: A,B,C,D) ANOVAs with normative valence and arousal ratings. For valence we observed a significant image valence effect ($F_{1,152}$=621.97, p<.001, ηp^2= .80), with unpleasant pictures rated as more unpleasant then the neutral ones (Tukey HSD test, p<.001). The set and the interaction between set and image category were not significant ($F_{1,152}$=0.34, p=.80, ηp^2= .01 and $F_{1,152}$=0.58, p=.63, ηp^2= .01, respectively). For arousal we observed a significant image category effect ($F_{1,152}$=543.18, p<.001, ηp^2= .78), with unpleasant pictures rated as higher in arousal than the neutral ones (Tukey HSD test, p<.001). Again, the set and the interaction between set and image valence were not significant ($F_{1,152}$=0.48, p=.69, ηp^2= .01 and $F_{1,152}$=1.26, p=.29, ηp^2= .02, respectively). A summary of the ratings can be found in Table S1.

Table S1. IAPS normative valence and arousal scores for the four sets of pictures used in the current study.

		Set A		Set B		Set C		Set D	
		Mean	SD	Mean	SD	Mean	SD	Mean	SD
**Valence**	Unpleasant	2.6	0.64	2.74	0.66	2.71	0.81	2.78	0.88
	Neutral	5.03	0.20	5.06	0.27	5.03	0.26	4.72	0.73
**Arousal**	Unpleasant	5.77	0.25	5.81	0.17	5.58	1.06	5.83	0.65
	Neutral	3.00	0.54	3.10	0.37	3.17	0.53	3.19	1.11

Notes. IAPS: International Affective Picture System. Picture reference number. **SET A.** *Unpleasant:* 1070, 1274, 2661, 2683, 2688, 3015, 3101, 3225, 6190, 6212, 6243, 6831, 7380, 9160, 9181, 9400, 9428, 9611, 9903, 9925. *Neutral:* 2102, 2214, 2305, 2397, 2512, 2595, 2840, 2890, 5500, 5531, 6150, 7006, 7025, 7037, 7050, 7056, 7080, 7175, 7233, 7547. **SET B.** *Unpleasant:* 1019,1090, 1220, 2703, 2981, 3016, 3062, 3180, 6200, 6244, 6555, 6838, 9040, 9405, 9423, 9495, 9500, 9622, 9902, 9920. *Neutral*: 2191, 2210, 2383, 2441, 2480, 2499, 2745.1, 5510, 5533, 7000, 7009, 7030, 7036, 7052, 7057, 7090, 7185, 7235, 7493, 7546. **SET C.** *Unpleasant:* 1040, 1110, 1205, 2800, 3051, 3168, 3216, 6211, 6410, 6415, 6940, 8230, 9420, 9424, 9520, 9523, 9570, 9621, 9901, 9911. *Neutral*: 2200, 2272, 2385, 2393, 2493, 2495, 2749, 5520, 5534, 5731, 7002, 7010, 7035, 7053, 7058, 7100, 7186, 7500, 7550, 7705. **SET D.** *Unpleasant:* 1051, 1113, 1200, 2352.2, 3005.1, 3160, 3350, 6022, 6242, 6570, 7359, 9120, 9140, 9254, 9427, 9433, 9571, 9620, 9900, 9910. *Neutral*: 2190, 2215, 2396, 2516, 2570, 2850, 2870, 5471, 5530, 7004, 7020, 7038, 7055, 7059, 7150, 7187, 7491, 7595, 7950, 9070.

Sleep Diary data

Table S2 summarizes the sleep parameters extracted from the sleep diaries.

Table S2. Sleep diary parameters of the sample.

	LDS		HDS				
	**Mean**	**SD**	**Mean**	**SD**	**$t_{(45)}$**	***p***	**Cohen's d**
Sleep Parameters							
Bed Time (hh:mm)	1:03	00:58	1:16	01:03	-1.07	.287	-0.32
Wake Time (hh:mm)	8:40	00:49	8:51	00:50	-0.74	.462	-0.22
Time in Bed (min)	468.42	42.16	461.87	47.45	0.49	.621	0.15
Sleep Latency (min)	15.60	8.87	18.99	17.31	-0.89	.375	-0.27
Total Sleep Time (min)	436.18	42.15	427.82	35.39	0.71	.484	0.16
Sleep Efficiency (%)	93.60	5.69	93.44	5.12	0.09	.922	0.03

Notes. WASO: Wake After Sleep Onset.

No differences were observed for sleep parameters extracted from the sleep diaries. Comparing sleep parameters for the objective (actigraphy) and subjective (sleep diary) measures

and the two groups using a 2 ×2 mixed ANOVA (Instrument as within-subjects and Group as between-subjects factors), we observed a significant effect of the Instrument for time in bed ($F_{1, 46}$=17.89, *p*<.001, ηp^2=.28), total sleep time ($F_{1, 46}$=56.55, *p*<.001, ηp^2=.55), sleep latency ($F_{1, 46}$=17.33, *p*<.001, ηp^2=.27), and sleep efficiency ($F_{1, 46}$=31.04, *p*<.001, ηp^2=.41), with the sleep diary constantly overestimating time in bed, total sleep time, sleep latency and sleep efficiency. This is likely due to the tendency of the participants to round up the reported time (e.g., instead of 7 hours and 20 minutes spent in bed they tend to report 7 hours and 30 minutes) and to report a total sleep time similar to the time in bed. No differences between the two groups (all *p's* > .10) or interaction between Group and Instrument were observed (all *p's* > .26).

Behavioral performance of the whole group correcting for BDI-II scores

Analyzing the behavioral data with a 2x2 ANCOVA, with Session (Immediate and Delayed Recognition Session) and category of the stimuli (Unpleasant and Neutral) as within-subjects factors and BDI-II scores as covariate, we observed similar results to what we reported in the main manuscript when dividing the sample into high and low depressive symptoms groups.

Memory performance

For the memory discrimination index (*d*'), we observed a significant Session main effect ($F_{1,46}$=74.22 *p*<.001, ηp^2=.62), with post-hoc tests indicating a lower *d'* for both the stimuli at the delayed compared to the immediate recognition session (*p*<.001). We also observed a trend for the Session × Category interaction ($F_{1,46}$=3.00, *p*=.090, ηp^2=.06), with higher memory discrimination index for neutral stimuli relative to unpleasant pictures at the immediate (*p*=.019), but not at the delayed recognition session (*p*=.449), indicating a lower forgetting for the unpleasant stimuli.

The analysis of the hit rate showed only a significant main Session effect ($F_{1,46}$=39.19 p<.001, ηp^2=.46), with a decreased hit rate in the delayed session compared to the immediate one (Figure 4a).

The analysis of the false alarm rate showed only a trend for a Session effect ($F_{1,46}$ = 2.08, p=.086, ηp^2=.06), with an increased false alarm rate in the delayed session compared to the immediate one.

Affective ratings

For the affective ratings, we analyzed the data with separate 2×2 ANCOVAs with Category (Unpleasant, Neutral) and Session (Encoding, Immediate or Delayed Recognition Session) as within-subject factors and BDI-II scores as covariate.

Immediate Recognition Test

The analysis of the valence ratings showed a significant Category main effect ($F_{1,46}$=148.20, p<.001, ηp^2= .76), with higher valence ratings for neutral than unpleasant pictures, and a significant Session main effect ($F_{1,46}$=4.36, p=.042, ηp^2=.08), with higher valence scores in the immediate recognition session compared to the encoding session.

The analysis of the arousal ratings showed a significant Category main effect ($F_{1,46}$=77.52, p<.001, ηp^2= .63), with higher ratings for unpleasant than neutral pictures, and a significant Session × BDI-II score interaction ($F_{1,46}$=4.26, p<.001, ηp^2=.08).

Delayed Recognition Test

The analysis of the valence ratings showed a significant Session main effect ($F_{1, 46}$=5.76, *p*=.021, ηp^2 = .11), with an increased valence in the delayed recognition session compared to the encoding session, and a significant Category main effect ($F_{1,46}$=140.33, p<.001, ηp^2=.75), with a significantly higher valence for the unpleasant stimuli compared to the neutral ones (p<.001). We also observed a trend for the Session × BDI-II score interaction ($F_{1,46}$=3.92, *p*=.054, ηp^2=.08). The analysis of the arousal ratings showed a significant Category main effect ($F_{1,46}$=89.79, *p*<.001, ηp^2=.66), with again higher arousal ratings for unpleasant than neutral pictures, and a significant Category × BDI-II score interaction ($F_{1,46}$=5.92, *p*=.019, ηp^2=.11). No other significant differences were observed.

Correlation between BDI-II and behavioral measures

The BDI-II scores for the whole sample showed no association with the memory performance measures (all *r's*<.09, *p's*>.50). We found a trend for significant associations between BDI and arousal ratings of neutral pictures at the encoding session (*r*=.27, *p*=.068) and valence ratings for unpleasant pictures at the encoding session (*r*=.25, *p*=.092). All the other correlations were non-significant (all *r's*<.22, *p's*>.13). Regarding the sleep data, the only significant correlation was between BDI-II scores and sleep latency (*r*=.33, *p*=.020), which reflects the increased sleep latency we reported in the HSD group in Table 1.

Correlational analyses on performance and sleep parameters

We observed a negative association between sleep efficiency (SE) and memory consolidation of unpleasant stimuli over time in the whole group (*r*=-.32, *p*=.026, Figure S1),

which did not change by correcting this correlation for the BDI-II scores (partial correlation with BDI-II as covariate, r=-.33, p=.026).

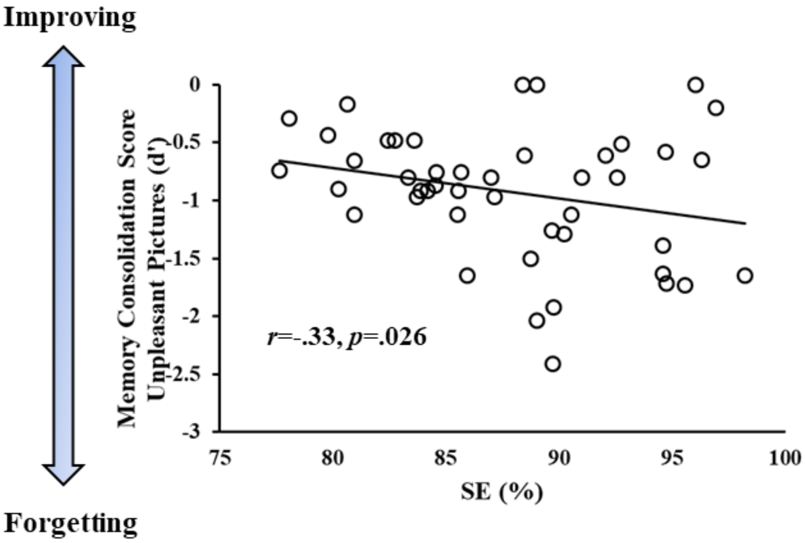

Figure S1. Memory consolidation score (d’ of the delayed minus d’ of the immediate recognition session) of the unpleasant pictures as a function of sleep efficiency (SE) for the whole sample.
